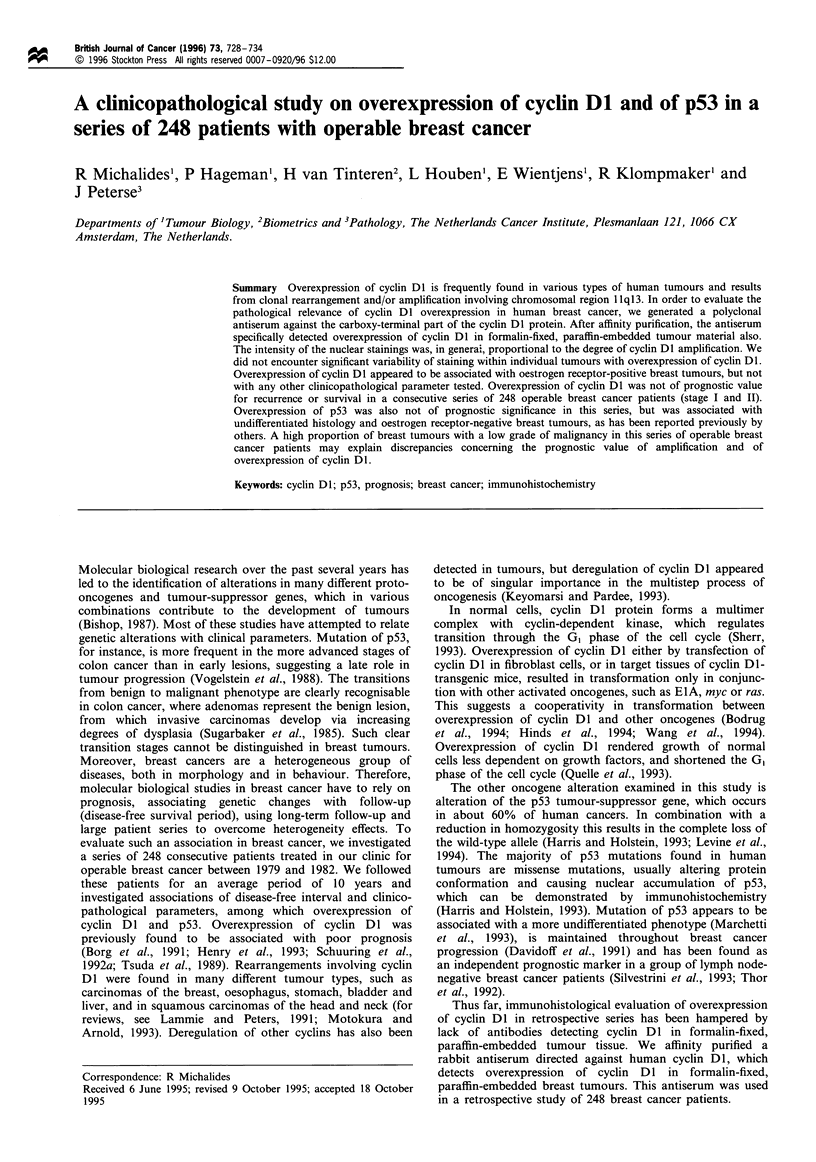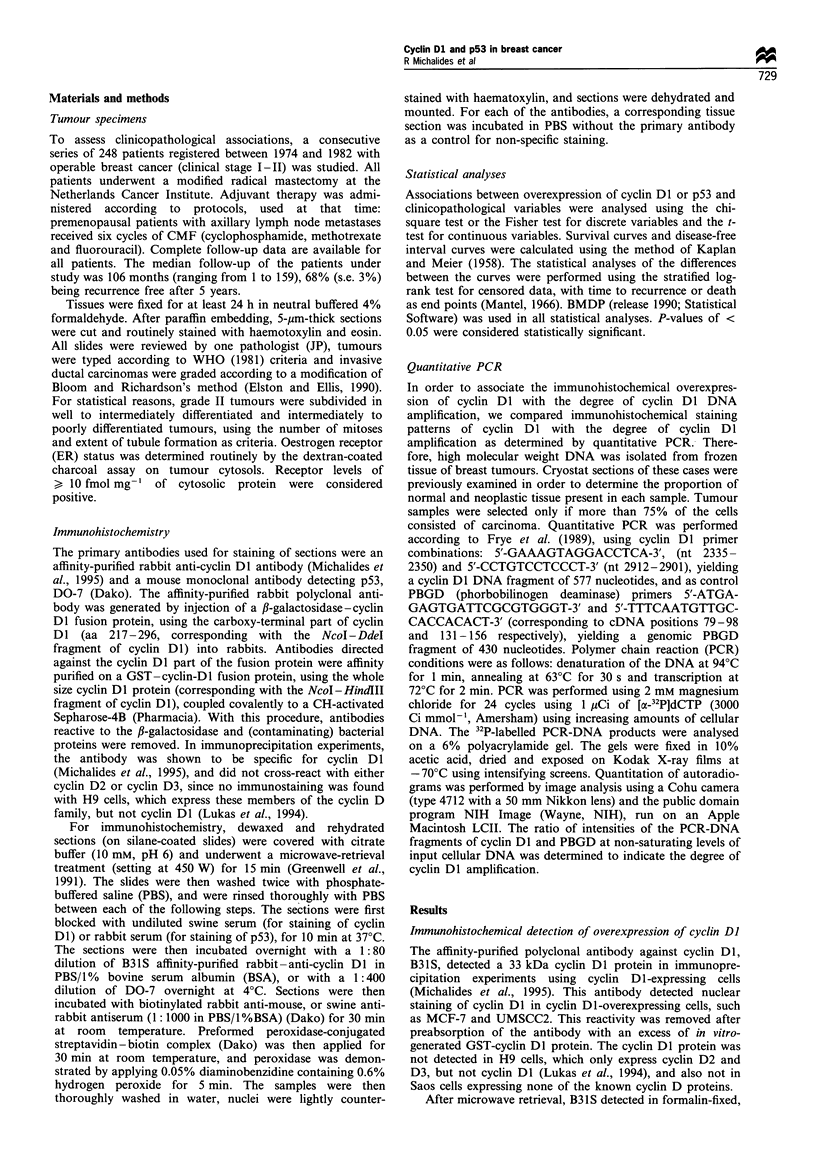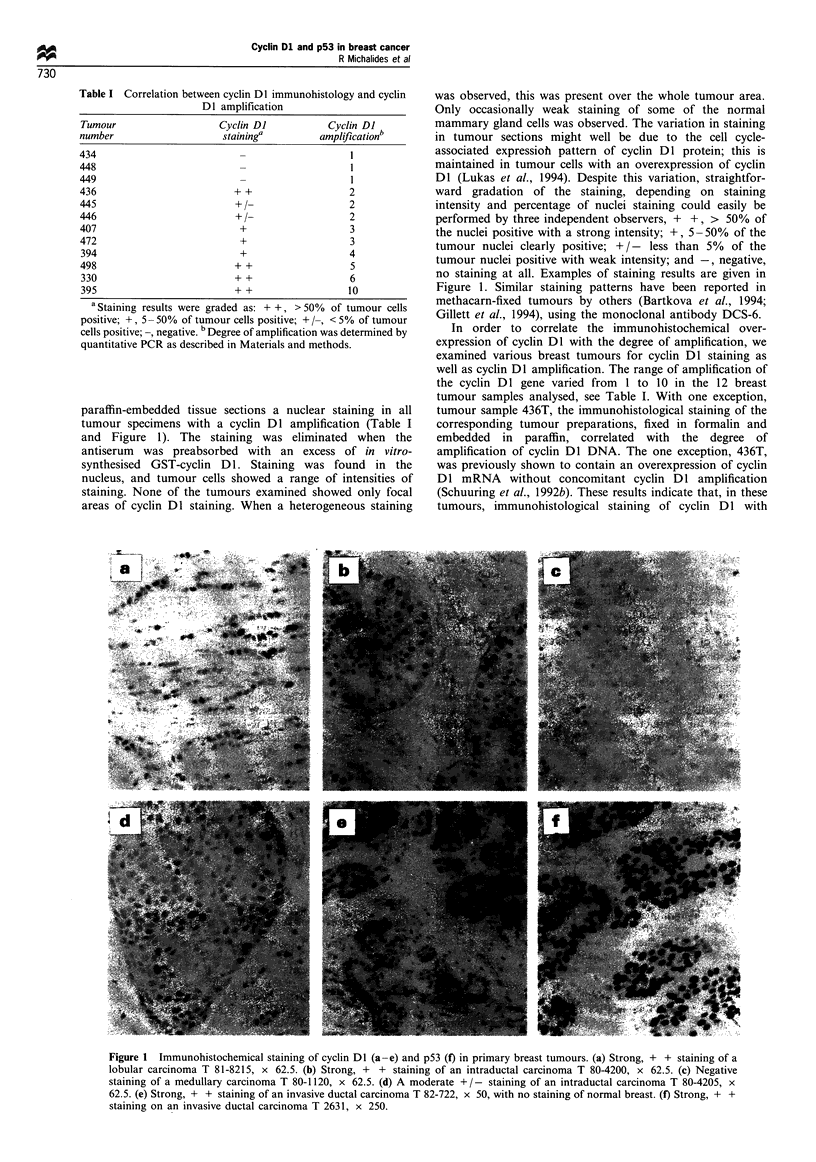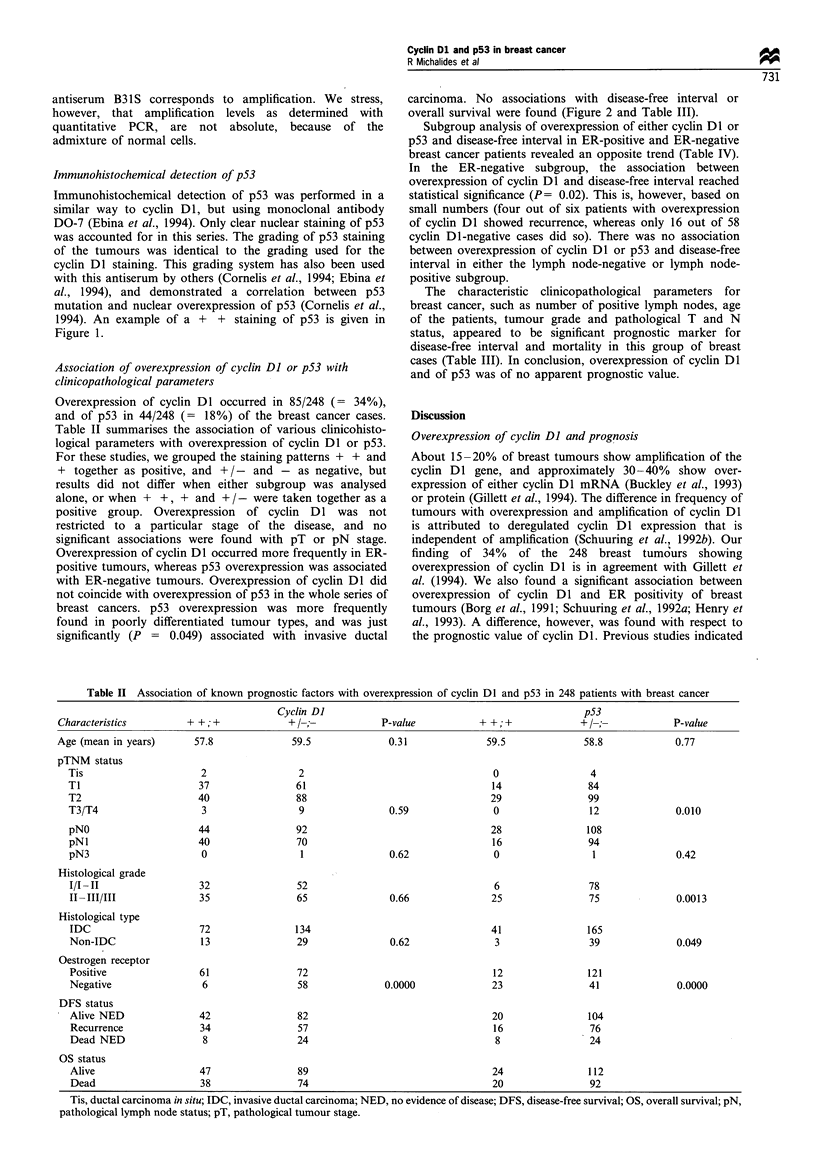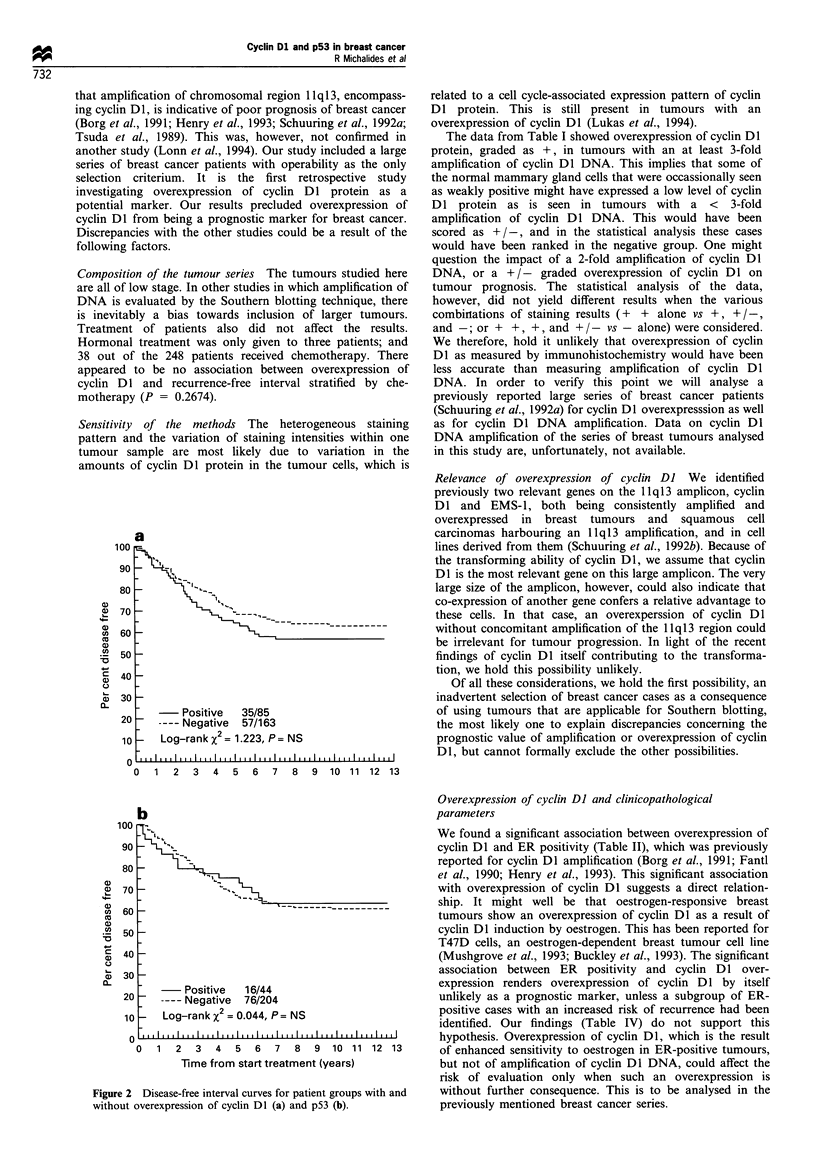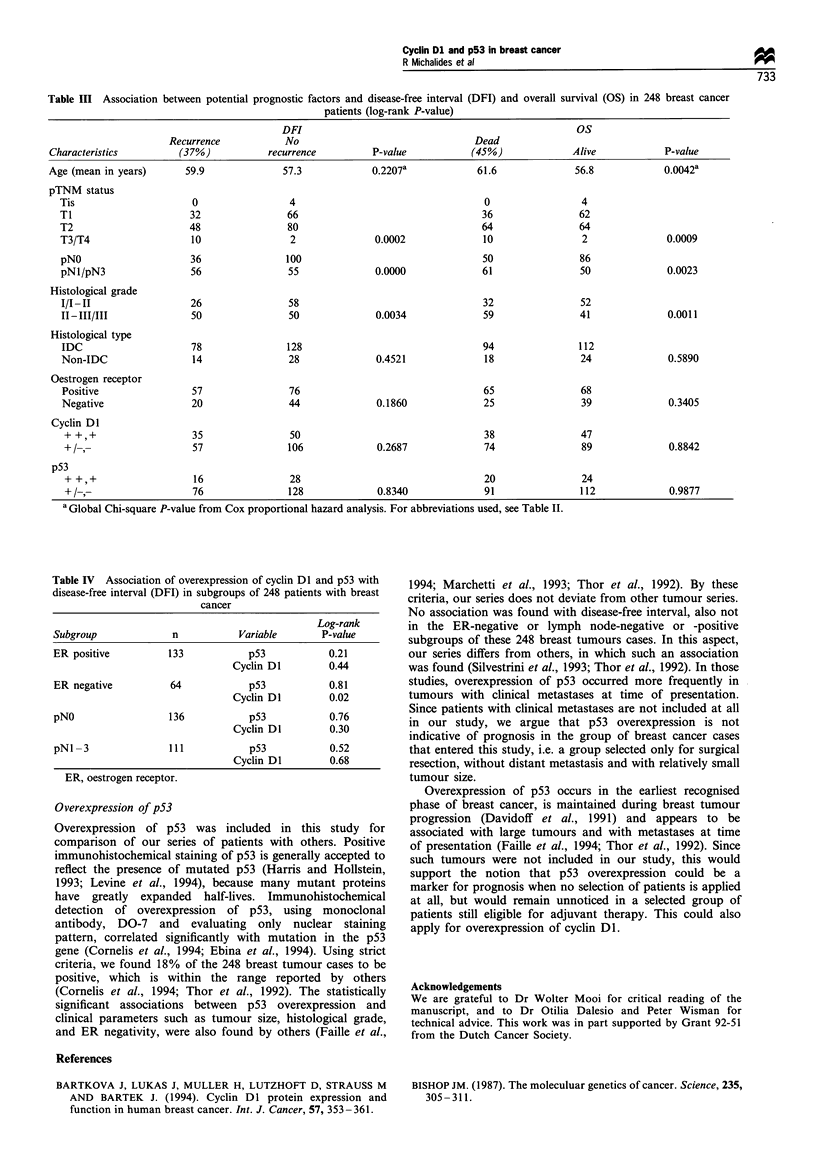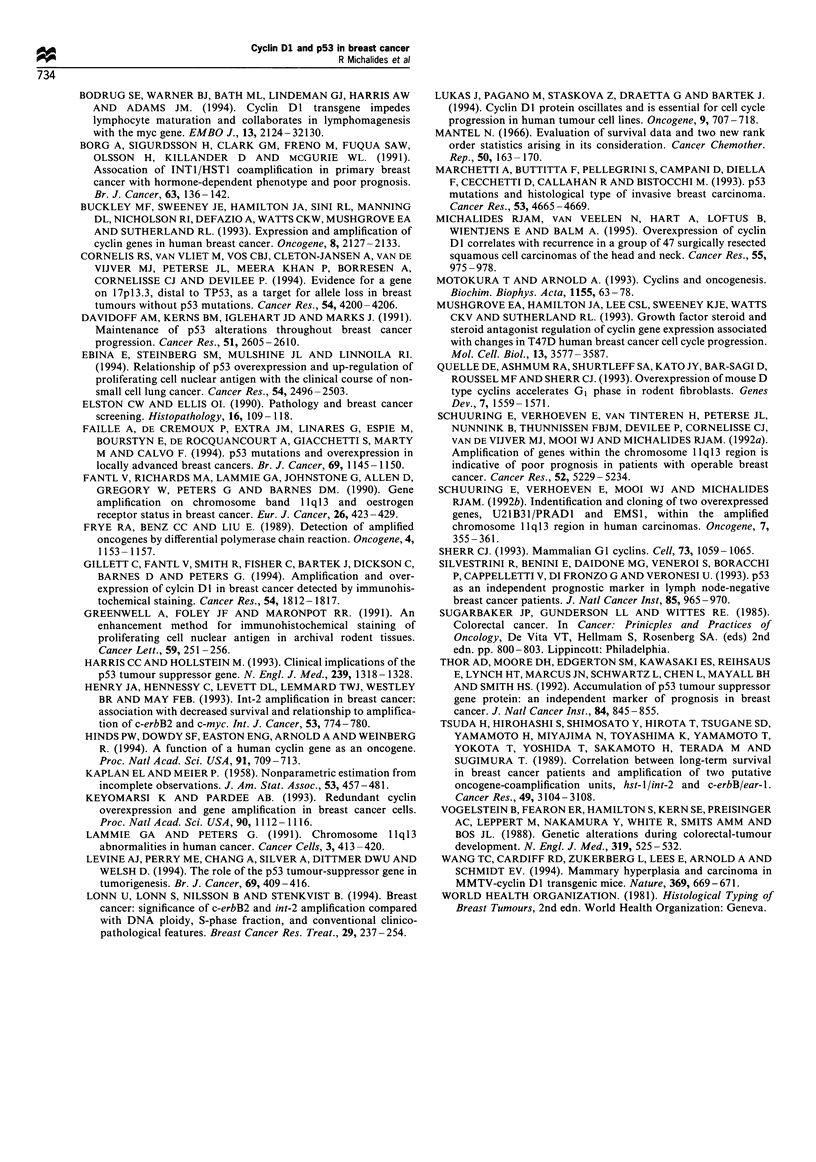# A clinicopathological study on overexpression of cyclin D1 and of p53 in a series of 248 patients with operable breast cancer.

**DOI:** 10.1038/bjc.1996.128

**Published:** 1996-03

**Authors:** R. Michalides, P. Hageman, H. van Tinteren, L. Houben, E. Wientjens, R. Klompmaker, J. Peterse

**Affiliations:** Department of Tumour Biology, The Netherlands Cancer Institute, Amsterdam.

## Abstract

**Images:**


British Journal of Cancer (1996) 73, 728–734
© 1996 Stockton Press All rights reserved 0007–0920/96 $12.00

### A clinicopathological study on overexpression of cyclin D1 and of p53 in a series of 248 patients with operable breast cancer

R Michalides[1], P Hageman[1], H van Tinteren[2], L Houben[1], E Wientjens[1], R Klompmaker[1] and J Peterse[3]

*Departments of [1]Tumour Biology, [2]Biometrics and [3]Pathology, The Netherlands Cancer Institute, Plesmanlaan 121, 1066 CX Amsterdam, The Netherlands.*

**Summary** Overexpression of cyclin D1 is frequently found in various types of human tumours and results from clonal rearrangement and/or amplification involving chromosomal region 11q13. In order to evaluate the pathological relevance of cyclin D1 overexpression in human breast cancer, we generated a polyclonal antiserum against the carboxy-terminal part of the cyclin D1 protein. After affinity purification, the antiserum specifically detected overexpression of cyclin D1 in formalin-fixed, paraffin-embedded tumour material also. The intensity of the nuclear stainings was, in general, proportional to the degree of cyclin D1 amplification. We did not encounter significant variability of staining within individual tumours with overexpression of cyclin D1. Overexpression of cyclin D1 appeared to be associated with oestrogen receptor-positive breast tumours, but not with any other clinicopathological parameter tested. Overexpression of cyclin D1 was not of prognostic value for recurrence or survival in a consecutive series of 248 operable breast cancer patients (stage I and II). Overexpression of p53 was also not of prognostic significance in this series, but was associated with undifferentiated histology and oestrogen receptor-negative breast tumours, as has been reported previously by others. A high proportion of breast tumours with a low grade of malignancy in this series of operable breast cancer patients may explain discrepancies concerning the prognostic value of amplification and of overexpression of cyclin D1.

**Keywords:** cyclin D1; p53, prognosis; breast cancer; immunohistochemistry

Molecular biological research over the past several years has led to the identification of alterations in many different proto-oncogenes and tumour-suppressor genes, which in various combinations contribute to the development of tumours (Bishop, 1987). Most of these studies have attempted to relate genetic alterations with clinical parameters. Mutation of p53, for instance, is more frequent in the more advanced stages of colon cancer than in early lesions, suggesting a late role in tumour progression (Vogelstein *et al.*, 1988). The transitions from benign to malignant phenotype are clearly recognisable in colon cancer, where adenomas represent the benign lesion, from which invasive carcinomas develop via increasing degrees of dysplasia (Sugarbaker *et al.*, 1985). Such clear transition stages cannot be distinguished in breast tumours. Moreover, breast cancers are a heterogeneous group of diseases, both in morphology and in behaviour. Therefore, molecular biological studies in breast cancer have to rely on prognosis, associating genetic changes with follow-up (disease-free survival period), using long-term follow-up and large patient series to overcome heterogeneity effects. To evaluate such an association in breast cancer, we investigated a series of 248 consecutive patients treated in our clinic for operable breast cancer between 1979 and 1982. We followed these patients for an average period of 10 years and investigated associations of disease-free interval and clinicopathological parameters, among which overexpression of cyclin D1 and p53. Overexpression of cyclin D1 was previously found to be associated with poor prognosis (Borg *et al.*, 1991; Henry *et al.*, 1993; Schuuring *et al.*, 1992*a*; Tsuda *et al.*, 1989). Rearrangements involving cyclin D1 were found in many different tumour types, such as carcinomas of the breast, oesophagus, stomach, bladder and liver, and in squamous carcinomas of the head and neck (for reviews, see Lammie and Peters, 1991; Motokura and Arnold, 1993). Deregulation of other cyclins has also been detected in tumours, but deregulation of cyclin D1 appeared to be of singular importance in the multistep process of oncogenesis (Keyomarsi and Pardee, 1993).

In normal cells, cyclin D1 protein forms a multimer complex with cyclin-dependent kinase, which regulates transition through the $G_1$ phase of the cell cycle (Sherr, 1993). Overexpression of cyclin D1 either by transfection of cyclin D1 in fibroblast cells, or in target tissues of cyclin D1-transgenic mice, resulted in transformation only in conjunction with other activated oncogenes, such as E1A, *myc* or *ras*. This suggests a cooperativity in transformation between overexpression of cyclin D1 and other oncogenes (Bodrug *et al.*, 1994; Hinds *et al.*, 1994; Wang *et al.*, 1994). Overexpression of cyclin D1 rendered growth of normal cells less dependent on growth factors, and shortened the $G_1$ phase of the cell cycle (Quelle *et al.*, 1993).

The other oncogene alteration examined in this study is alteration of the p53 tumour-suppressor gene, which occurs in about 60% of human cancers. In combination with a reduction in homozygosity this results in the complete loss of the wild-type allele (Harris and Holstein, 1993; Levine *et al.*, 1994). The majority of p53 mutations found in human tumours are missense mutations, usually altering protein conformation and causing nuclear accumulation of p53, which can be demonstrated by immunohistochemistry (Harris and Holstein, 1993). Mutation of p53 appears to be associated with a more undifferentiated phenotype (Marchetti *et al.*, 1993), is maintained throughout breast cancer progression (Davidoff *et al.*, 1991) and has been found as an independent prognostic marker in a group of lymph node-negative breast cancer patients (Silvestrini *et al.*, 1993; Thor *et al.*, 1992).

Thus far, immunohistological evaluation of overexpression of cyclin D1 in retrospective series has been hampered by lack of antibodies detecting cyclin D1 in formalin-fixed, paraffin-embedded tumour tissue. We affinity purified a rabbit antiserum directed against human cyclin D1, which detects overexpression of cyclin D1 in formalin-fixed, paraffin-embedded breast tumours. This antiserum was used in a retrospective study of 248 breast cancer patients.

Correspondence: R Michalides
Received 6 June 1995; revised 9 October 1995; accepted 18 October 1995

### Materials and methods

*Tumour specimens*

To assess clinicopathological associations, a consecutive series of 248 patients registered between 1974 and 1982 with operable breast cancer (clinical stage I–II) was studied. All patients underwent a modified radical mastectomy at the Netherlands Cancer Institute. Adjuvant therapy was administered according to protocols, used at that time: premenopausal patients with axillary lymph node metastases received six cycles of CMF (cyclophosphamide, methotrexate and fluorouracil). Complete follow-up data are available for all patients. The median follow-up of the patients under study was 106 months (ranging from 1 to 159), 68% (s.e. 3%) being recurrence free after 5 years.

Tissues were fixed for at least 24 h in neutral buffered 4% formaldehyde. After paraffin embedding, 5-$\mu$m-thick sections were cut and routinely stained with haemotoxylin and eosin. All slides were reviewed by one pathologist (JP), tumours were typed according to WHO (1981) criteria and invasive ductal carcinomas were graded according to a modification of Bloom and Richardson's method (Elston and Ellis, 1990). For statistical reasons, grade II tumours were subdivided in well to intermediately differentiated and intermediately to poorly differentiated tumours, using the number of mitoses and extent of tubule formation as criteria. Oestrogen receptor (ER) status was determined routinely by the dextran-coated charcoal assay on tumour cytosols. Receptor levels of $\geqslant$ 10 fmol mg$^{-1}$ of cytosolic protein were considered positive.

*Immunohistochemistry*

The primary antibodies used for staining of sections were an affinity-purified rabbit anti-cyclin D1 antibody (Michalides *et al.*, 1995) and a mouse monoclonal antibody detecting p53, DO-7 (Dako). The affinity-purified rabbit polyclonal antibody was generated by injection of a $\beta$-galactosidase–cyclin D1 fusion protein, using the carboxy-terminal part of cyclin D1 (aa 217–296, corresponding with the *Nco*I–*Dde*I fragment of cyclin D1) into rabbits. Antibodies directed against the cyclin D1 part of the fusion protein were affinity purified on a GST–cyclin-D1 fusion protein, using the whole size cyclin D1 protein (corresponding with the *Nco*I–*Hind*III fragment of cyclin D1), coupled covalently to a CH-activated Sepharose-4B (Pharmacia). With this procedure, antibodies reactive to the $\beta$-galactosidase and (contaminating) bacterial proteins were removed. In immunoprecipitation experiments, the antibody was shown to be specific for cyclin D1 (Michalides *et al.*, 1995), and did not cross-react with either cyclin D2 or cyclin D3, since no immunostaining was found with H9 cells, which express these members of the cyclin D family, but not cyclin D1 (Lukas *et al.*, 1994).

For immunohistochemistry, dewaxed and rehydrated sections (on silane-coated slides) were covered with citrate buffer (10 mM, pH 6) and underwent a microwave-retrieval treatment (setting at 450 W) for 15 min (Greenwell *et al.*, 1991). The slides were then washed twice with phosphate-buffered saline (PBS), and were rinsed thoroughly with PBS between each of the following steps. The sections were first blocked with undiluted swine serum (for staining of cyclin D1) or rabbit serum (for staining of p53), for 10 min at 37°C. The sections were then incubated overnight with a 1:80 dilution of B31S affinity-purified rabbit–anti-cyclin D1 in PBS/1% bovine serum albumin (BSA), or with a 1:400 dilution of DO-7 overnight at 4°C. Sections were then incubated with biotinylated rabbit anti-mouse, or swine anti-rabbit antiserum (1:1000 in PBS/1%BSA) (Dako) for 30 min at room temperature. Preformed peroxidase-conjugated streptavidin–biotin complex (Dako) was then applied for 30 min at room temperature, and peroxidase was demonstrated by applying 0.05% diaminobenzidine containing 0.6% hydrogen peroxide for 5 min. The samples were then thoroughly washed in water, nuclei were lightly counter-stained with haematoxylin, and sections were dehydrated and mounted. For each of the antibodies, a corresponding tissue section was incubated in PBS without the primary antibody as a control for non-specific staining.

*Statistical analyses*

Associations between overexpression of cyclin D1 or p53 and clinicopathological variables were analysed using the chi-square test or the Fisher test for discrete variables and the *t*-test for continuous variables. Survival curves and disease-free interval curves were calculated using the method of Kaplan and Meier (1958). The statistical analyses of the differences between the curves were performed using the stratified log-rank test for censored data, with time to recurrence or death as end points (Mantel, 1966). BMDP (release 1990; Statistical Software) was used in all statistical analyses. *P*-values of < 0.05 were considered statistically significant.

*Quantitative PCR*

In order to associate the immunohistochemical overexpression of cyclin D1 with the degree of cyclin D1 DNA amplification, we compared immunohistochemical staining patterns of cyclin D1 with the degree of cyclin D1 amplification as determined by quantitative PCR. Therefore, high molecular weight DNA was isolated from frozen tissue of breast tumours. Cryostat sections of these cases were previously examined in order to determine the proportion of normal and neoplastic tissue present in each sample. Tumour samples were selected only if more than 75% of the cells consisted of carcinoma. Quantitative PCR was performed according to Frye *et al.* (1989), using cyclin D1 primer combinations: 5′-GAAAGTAGGACCTCA-3′, (nt 2335–2350) and 5′-CCTGTCCTCCCT-3′ (nt 2912–2901), yielding a cyclin D1 DNA fragment of 577 nucleotides, and as control PBGD (phorbobilinogen deaminase) primers 5′-ATGAGAGTGATTCGCGTGGGT-3′ and 5′-TTTCAATGTTGCCACCACACT-3′ (corresponding to cDNA positions 79–98 and 131–156 respectively), yielding a genomic PBGD fragment of 430 nucleotides. Polymer chain reaction (PCR) conditions were as follows: denaturation of the DNA at 94°C for 1 min, annealing at 63°C for 30 s and transcription at 72°C for 2 min. PCR was performed using 2 mM magnesium chloride for 24 cycles using 1 $\mu$Ci of [$\alpha$-$^{32}$P]dCTP (3000 Ci mmol$^{-1}$, Amersham) using increasing amounts of cellular DNA. The $^{32}$P-labelled PCR-DNA products were analysed on a 6% polyacrylamide gel. The gels were fixed in 10% acetic acid, dried and exposed on Kodak X-ray films at −70°C using intensifying screens. Quantitation of autoradiograms was performed by image analysis using a Cohu camera (type 4712 with a 50 mm Nikkon lens) and the public domain program NIH Image (Wayne, NIH), run on an Apple Macintosh LCII. The ratio of intensities of the PCR-DNA fragments of cyclin D1 and PBGD at non-saturating levels of input cellular DNA was determined to indicate the degree of cyclin D1 amplification.

### Results

*Immunohistochemical detection of overexpression of cyclin D1*

The affinity-purified polyclonal antibody against cyclin D1, B31S, detected a 33 kDa cyclin D1 protein in immunoprecipitation experiments using cyclin D1-expressing cells (Michalides *et al.*, 1995). This antibody detected nuclear staining of cyclin D1 in cyclin D1-overexpressing cells, such as MCF-7 and UMSCC2. This reactivity was removed after preabsorption of the antibody with an excess of *in vitro*-generated GST-cyclin D1 protein. The cyclin D1 protein was not detected in H9 cells, which only express cyclin D2 and D3, but not cyclin D1 (Lukas *et al.*, 1994), and also not in Saos cells expressing none of the known cyclin D proteins.

After microwave retrieval, B31S detected in formalin-fixed,

**Table I** Correlation between cyclin D1 immunohistology and cyclin D1 amplification

| *Tumour number* | *Cyclin D1 staining*[a] | *Cyclin D1 amplification*[b] |
|---|---|---|
| 434 | – | 1 |
| 448 | – | 1 |
| 449 | – | 1 |
| 436 | + + | 2 |
| 445 | +/– | 2 |
| 446 | +/– | 2 |
| 407 | + | 3 |
| 472 | + | 3 |
| 394 | + | 4 |
| 498 | + + | 5 |
| 330 | + + | 6 |
| 395 | + + | 10 |

[a] Staining results were graded as: + +, > 50% of tumour cells positive; +, 5–50% of tumour cells positive; +/–, < 5% of tumour cells positive; –, negative. [b] Degree of amplification was determined by quantitative PCR as described in Materials and methods.

paraffin-embedded tissue sections a nuclear staining in all tumour specimens with a cyclin D1 amplification (Table I and Figure 1). The staining was eliminated when the antiserum was preabsorbed with an excess of *in vitro*-synthesised GST-cyclin D1. Staining was found in the nucleus, and tumour cells showed a range of intensities of staining. None of the tumours examined showed only focal areas of cyclin D1 staining. When a heterogeneous staining was observed, this was present over the whole tumour area. Only occasionally weak staining of some of the normal mammary gland cells was observed. The variation in staining in tumour sections might well be due to the cell cycle-associated expression pattern of cyclin D1 protein; this is maintained in tumour cells with an overexpression of cyclin D1 (Lukas *et al.*, 1994). Despite this variation, straightforward gradation of the staining, depending on staining intensity and percentage of nuclei staining could easily be performed by three independent observers, + +, > 50% of the nuclei positive with a strong intensity; +, 5–50% of the tumour nuclei clearly positive; +/– less than 5% of the tumour nuclei positive with weak intensity; and –, negative, no staining at all. Examples of staining results are given in Figure 1. Similar staining patterns have been reported in methacarn-fixed tumours by others (Bartkova *et al.*, 1994; Gillett *et al.*, 1994), using the monoclonal antibody DCS-6.

In order to correlate the immunohistochemical over-expression of cyclin D1 with the degree of amplification, we examined various breast tumours for cyclin D1 staining as well as cyclin D1 amplification. The range of amplification of the cyclin D1 gene varied from 1 to 10 in the 12 breast tumour samples analysed, see Table I. With one exception, tumour sample 436T, the immunohistological staining of the corresponding tumour preparations, fixed in formalin and embedded in paraffin, correlated with the degree of amplification of cyclin D1 DNA. The one exception, 436T, was previously shown to contain an overexpression of cyclin D1 mRNA without concomitant cyclin D1 amplification (Schuuring *et al.*, 1992*b*). These results indicate that, in these tumours, immunohistological staining of cyclin D1 with

**Figure 1** Immunohistochemical staining of cyclin D1 (**a**–**e**) and p53 (**f**) in primary breast tumours. (**a**) Strong, + + staining of a lobular carcinoma T 81-8215, × 62.5. (**b**) Strong, + + staining of an intraductal carcinoma T 80-4200, × 62.5. (**c**) Negative staining of a medullary carcinoma T 80-1120, × 62.5. (**d**) A moderate +/– staining of an intraductal carcinoma T 80-4205, × 62.5. (**e**) Strong, + + staining of an invasive ductal carcinoma T 82-722, × 50, with no staining of normal breast. (**f**) Strong, + + staining on an invasive ductal carcinoma T 2631, × 250.

antiserum B31S corresponds to amplification. We stress, however, that amplification levels as determined with quantitative PCR, are not absolute, because of the admixture of normal cells.

*Immunohistochemical detection of p53*

Immunohistochemical detection of p53 was performed in a similar way to cyclin D1, but using monoclonal antibody DO-7 (Ebina *et al.*, 1994). Only clear nuclear staining of p53 was accounted for in this series. The grading of p53 staining of the tumours was identical to the grading used for the cyclin D1 staining. This grading system has also been used with this antiserum by others (Cornelis *et al.*, 1994; Ebina *et al.*, 1994), and demonstrated a correlation between p53 mutation and nuclear overexpression of p53 (Cornelis *et al.*, 1994). An example of a + + staining of p53 is given in Figure 1.

*Association of overexpression of cyclin D1 or p53 with clinicopathological parameters*

Overexpression of cyclin D1 occurred in 85/248 (= 34%), and of p53 in 44/248 (= 18%) of the breast cancer cases. Table II summarises the association of various clinicohistological parameters with overexpression of cyclin D1 or p53. For these studies, we grouped the staining patterns + + and + together as positive, and +/− and − as negative, but results did not differ when either subgroup was analysed alone, or when + +, + and +/− were taken together as a positive group. Overexpression of cyclin D1 was not restricted to a particular stage of the disease, and no significant associations were found with pT or pN stage. Overexpression of cyclin D1 occurred more frequently in ER-positive tumours, whereas p53 overexpression was associated with ER-negative tumours. Overexpression of cyclin D1 did not coincide with overexpression of p53 in the whole series of breast cancers. p53 overexpression was more frequently found in poorly differentiated tumour types, and was just significantly ($P$ = 0.049) associated with invasive ductal carcinoma. No associations with disease-free interval or overall survival were found (Figure 2 and Table III).

Subgroup analysis of overexpression of either cyclin D1 or p53 and disease-free interval in ER-positive and ER-negative breast cancer patients revealed an opposite trend (Table IV). In the ER-negative subgroup, the association between overexpression of cyclin D1 and disease-free interval reached statistical significance ($P$= 0.02). This is, however, based on small numbers (four out of six patients with overexpression of cyclin D1 showed recurrence, whereas only 16 out of 58 cyclin D1-negative cases did so). There was no association between overexpression of cyclin D1 or p53 and disease-free interval in either the lymph node-negative or lymph node-positive subgroup.

The characteristic clinicopathological parameters for breast cancer, such as number of positive lymph nodes, age of the patients, tumour grade and pathological T and N status, appeared to be significant prognostic marker for disease-free interval and mortality in this group of breast cases (Table III). In conclusion, overexpression of cyclin D1 and of p53 was of no apparent prognostic value.

### Discussion

*Overexpression of cyclin D1 and prognosis*

About 15–20% of breast tumours show amplification of the cyclin D1 gene, and approximately 30–40% show overexpression of either cyclin D1 mRNA (Buckley *et al.*, 1993) or protein (Gillett *et al.*, 1994). The difference in frequency of tumours with overexpression and amplification of cyclin D1 is attributed to deregulated cyclin D1 expression that is independent of amplification (Schuuring *et al.*, 1992*b*). Our finding of 34% of the 248 breast tumours showing overexpression of cyclin D1 is in agreement with Gillett *et al.* (1994). We also found a significant association between overexpression of cyclin D1 and ER positivity of breast tumours (Borg *et al.*, 1991; Schuuring *et al.*, 1992*a*; Henry *et al.*, 1993). A difference, however, was found with respect to the prognostic value of cyclin D1. Previous studies indicated

**Table II** Association of known prognostic factors with overexpression of cyclin D1 and p53 in 248 patients with breast cancer

| *Characteristics* | Cyclin D1 + +;+ | Cyclin D1 +/–;– | P-*value* | p53 + +;+ | p53 +/–;– | P-*value* |
|---|---|---|---|---|---|---|
| Age (mean in years) | 57.8 | 59.5 | 0.31 | 59.5 | 58.8 | 0.77 |
| pTNM status | | | | | | |
| Tis | 2 | 2 | | 0 | 4 | |
| T1 | 37 | 61 | | 14 | 84 | |
| T2 | 40 | 88 | | 29 | 99 | |
| T3/T4 | 3 | 9 | 0.59 | 0 | 12 | 0.010 |
| pN0 | 44 | 92 | | 28 | 108 | |
| pN1 | 40 | 70 | | 16 | 94 | |
| pN3 | 0 | 1 | 0.62 | 0 | 1 | 0.42 |
| Histological grade | | | | | | |
| I/I–II | 32 | 52 | | 6 | 78 | |
| II–III/III | 35 | 65 | 0.66 | 25 | 75 | 0.0013 |
| Histological type | | | | | | |
| IDC | 72 | 134 | | 41 | 165 | |
| Non-IDC | 13 | 29 | 0.62 | 3 | 39 | 0.049 |
| Oestrogen receptor | | | | | | |
| Positive | 61 | 72 | | 12 | 121 | |
| Negative | 6 | 58 | 0.0000 | 23 | 41 | 0.0000 |
| DFS status | | | | | | |
| Alive NED | 42 | 82 | | 20 | 104 | |
| Recurrence | 34 | 57 | | 16 | 76 | |
| Dead NED | 8 | 24 | | 8 | 24 | |
| OS status | | | | | | |
| Alive | 47 | 89 | | 24 | 112 | |
| Dead | 38 | 74 | | 20 | 92 | |

Tis, ductal carcinoma *in situ*; IDC, invasive ductal carcinoma; NED, no evidence of disease; DFS, disease-free survival; OS, overall survival; pN, pathological lymph node status; pT, pathological tumour stage.

that amplification of chromosomal region 11q13, encompassing cyclin D1, is indicative of poor prognosis of breast cancer (Borg *et al.*, 1991; Henry *et al.*, 1993; Schuuring *et al.*, 1992*a*; Tsuda *et al.*, 1989). This was, however, not confirmed in another study (Lonn *et al.*, 1994). Our study included a large series of breast cancer patients with operability as the only selection criterium. It is the first retrospective study investigating overexpression of cyclin D1 protein as a potential marker. Our results precluded overexpression of cyclin D1 from being a prognostic marker for breast cancer. Discrepancies with the other studies could be a result of the following factors.

*Composition of the tumour series* The tumours studied here are all of low stage. In other studies in which amplification of DNA is evaluated by the Southern blotting technique, there is inevitably a bias towards inclusion of larger tumours. Treatment of patients also did not affect the results. Hormonal treatment was only given to three patients; and 38 out of the 248 patients received chemotherapy. There appeared to be no association between overexpression of cyclin D1 and recurrence-free interval stratified by chemotherapy ($P$ = 0.2674).

*Sensitivity of the methods* The heterogeneous staining pattern and the variation of staining intensities within one tumour sample are most likely due to variation in the amounts of cyclin D1 protein in the tumour cells, which is related to a cell cycle-associated expression pattern of cyclin D1 protein. This is still present in tumours with an overexpression of cyclin D1 (Lukas *et al.*, 1994).

The data from Table I showed overexpression of cyclin D1 protein, graded as +, in tumours with an at least 3-fold amplification of cyclin D1 DNA. This implies that some of the normal mammary gland cells that were occassionally seen as weakly positive might have expressed a low level of cyclin D1 protein as is seen in tumours with a < 3-fold amplification of cyclin D1 DNA. This would have been scored as +/−, and in the statistical analysis these cases would have been ranked in the negative group. One might question the impact of a 2-fold amplification of cyclin D1 DNA, or a +/− graded overexpression of cyclin D1 on tumour prognosis. The statistical analysis of the data, however, did not yield different results when the various combinations of staining results (+ + alone *vs* +, +/−, and −; or + +, +, and +/− *vs* − alone) were considered. We therefore, hold it unlikely that overexpression of cyclin D1 as measured by immunohistochemistry would have been less accurate than measuring amplification of cyclin D1 DNA. In order to verify this point we will analyse a previously reported large series of breast cancer patients (Schuuring *et al.*, 1992*a*) for cyclin D1 overexpresssion as well as for cyclin D1 DNA amplification. Data on cyclin D1 DNA amplification of the series of breast tumours analysed in this study are, unfortunately, not available.

*Relevance of overexpression of cyclin D1* We identified previously two relevant genes on the 11q13 amplicon, cyclin D1 and EMS-1, both being consistently amplified and overexpressed in breast tumours and squamous cell carcinomas harbouring an 11q13 amplification, and in cell lines derived from them (Schuuring *et al.*, 1992*b*). Because of the transforming ability of cyclin D1, we assume that cyclin D1 is the most relevant gene on this large amplicon. The very large size of the amplicon, however, could also indicate that co-expression of another gene confers a relative advantage to these cells. In that case, an overexperssion of cyclin D1 without concomitant amplification of the 11q13 region could be irrelevant for tumour progression. In light of the recent findings of cyclin D1 itself contributing to the transformation, we hold this possibility unlikely.

Of all these considerations, we hold the first possibility, an inadvertent selection of breast cancer cases as a consequence of using tumours that are applicable for Southern blotting, the most likely one to explain discrepancies concerning the prognostic value of amplification or overexpression of cyclin D1, but cannot formally exclude the other possibilities.

**Figure 2** Disease-free interval curves for patient groups with and without overexpression of cyclin D1 (**a**) and p53 (**b**).

### *Overexpression of cyclin D1 and clinicopathological parameters*

We found a significant association between overexpression of cyclin D1 and ER positivity (Table II), which was previously reported for cyclin D1 amplification (Borg *et al.*, 1991; Fantl *et al.*, 1990; Henry *et al.*, 1993). This significant association with overexpression of cyclin D1 suggests a direct relationship. It might well be that oestrogen-responsive breast tumours show an overexpression of cyclin D1 as a result of cyclin D1 induction by oestrogen. This has been reported for T47D cells, an oestrogen-dependent breast tumour cell line (Mushgrove *et al.*, 1993; Buckley *et al.*, 1993). The significant association between ER positivity and cyclin D1 overexpression renders overexpression of cyclin D1 by itself unlikely as a prognostic marker, unless a subgroup of ER-positive cases with an increased risk of recurrence had been identified. Our findings (Table IV) do not support this hypothesis. Overexpression of cyclin D1, which is the result of enhanced sensitivity to oestrogen in ER-positive tumours, but not of amplification of cyclin D1 DNA, could affect the risk of evaluation only when such an overexpression is without further consequence. This is to be analysed in the previously mentioned breast cancer series.

**Table III** Association between potential prognostic factors and disease-free interval (DFI) and overall survival (OS) in 248 breast cancer patients (log-rank *P*-value)

| *Characteristics* | DFI *Recurrence (37%)* | *No recurrence* | P-*value* | OS *Dead (45%)* | *Alive* | P-*value* |
|---|---|---|---|---|---|---|
| Age (mean in years) | 59.9 | 57.3 | 0.2207[a] | 61.6 | 56.8 | 0.0042[a] |
| pTNM status | | | | | | |
| Tis | 0 | 4 | | 0 | 4 | |
| T1 | 32 | 66 | | 36 | 62 | |
| T2 | 48 | 80 | | 64 | 64 | |
| T3/T4 | 10 | 2 | 0.0002 | 10 | 2 | 0.0009 |
| pN0 | 36 | 100 | | 50 | 86 | |
| pN1/pN3 | 56 | 55 | 0.0000 | 61 | 50 | 0.0023 |
| Histological grade | | | | | | |
| I/I-II | 26 | 58 | | 32 | 52 | |
| II-III/III | 50 | 50 | 0.0034 | 59 | 41 | 0.0011 |
| Histological type | | | | | | |
| IDC | 78 | 128 | | 94 | 112 | |
| Non-IDC | 14 | 28 | 0.4521 | 18 | 24 | 0.5890 |
| Oestrogen receptor | | | | | | |
| Positive | 57 | 76 | | 65 | 68 | |
| Negative | 20 | 44 | 0.1860 | 25 | 39 | 0.3405 |
| Cyclin D1 | | | | | | |
| + +,+ | 35 | 50 | | 38 | 47 | |
| +/-,- | 57 | 106 | 0.2687 | 74 | 89 | 0.8842 |
| p53 | | | | | | |
| + +,+ | 16 | 28 | | 20 | 24 | |
| +/-,- | 76 | 128 | 0.8340 | 91 | 112 | 0.9877 |

[a] Global Chi-square *P*-value from Cox proportional hazard analysis. For abbreviations used, see Table II.

**Table IV** Association of overexpression of cyclin D1 and p53 with disease-free interval (DFI) in subgroups of 248 patients with breast cancer

| *Subgroup* | n | *Variable* | *Log-rank* P-*value* |
|---|---|---|---|
| ER positive | 133 | p53 | 0.21 |
| | | Cyclin D1 | 0.44 |
| ER negative | 64 | p53 | 0.81 |
| | | Cyclin D1 | 0.02 |
| pN0 | 136 | p53 | 0.76 |
| | | Cyclin D1 | 0.30 |
| pN1-3 | 111 | p53 | 0.52 |
| | | Cyclin D1 | 0.68 |

ER, oestrogen receptor.

### *Overexpression of p53*

Overexpression of p53 was included in this study for comparison of our series of patients with others. Positive immunohistochemical staining of p53 is generally accepted to reflect the presence of mutated p53 (Harris and Hollstein, 1993; Levine *et al.*, 1994), because many mutant proteins have greatly expanded half-lives. Immunohistochemical detection of overexpression of p53, using monoclonal antibody, DO-7 and evaluating only nuclear staining pattern, correlated significantly with mutation in the p53 gene (Cornelis *et al.*, 1994; Ebina *et al.*, 1994). Using strict criteria, we found 18% of the 248 breast tumour cases to be positive, which is within the range reported by others (Cornelis *et al.*, 1994; Thor *et al.*, 1992). The statistically significant associations between p53 overexpression and clinical parameters such as tumour size, histological grade, and ER negativity, were also found by others (Faille *et al.*, 1994; Marchetti *et al.*, 1993; Thor *et al.*, 1992). By these criteria, our series does not deviate from other tumour series. No association was found with disease-free interval, also not in the ER-negative or lymph node-negative or -positive subgroups of these 248 breast tumours cases. In this aspect, our series differs from others, in which such an association was found (Silvestrini *et al.*, 1993; Thor *et al.*, 1992). In those studies, overexpression of p53 occurred more frequently in tumours with clinical metastases at time of presentation. Since patients with clinical metastases are not included at all in our study, we argue that p53 overexpression is not indicative of prognosis in the group of breast cancer cases that entered this study, i.e. a group selected only for surgical resection, without distant metastasis and with relatively small tumour size.

Overexpression of p53 occurs in the earliest recognised phase of breast cancer, is maintained during breast tumour progression (Davidoff *et al.*, 1991) and appears to be associated with large tumours and with metastases at time of presentation (Faille *et al.*, 1994; Thor *et al.*, 1992). Since such tumours were not included in our study, this would support the notion that p53 overexpression could be a marker for prognosis when no selection of patients is applied at all, but would remain unnoticed in a selected group of patients still eligible for adjuvant therapy. This could also apply for overexpression of cyclin D1.

**Acknowledgements**
We are grateful to Dr Wolter Mooi for critical reading of the manuscript, and to Dr Otilia Dalesio and Peter Wisman for technical advice. This work was in part supported by Grant 92-51 from the Dutch Cancer Society.

### References

BARTKOVA J, LUKAS J, MULLER H, LUTZHOFT D, STRAUSS M AND BARTEK J. (1994). Cyclin D1 protein expression and function in human breast cancer. *Int. J. Cancer*, **57**, 353-361.

BISHOP JM. (1987). The moleculuar genetics of cancer. *Science*, **235**, 305-311.

BODRUG SE, WARNER BJ, BATH ML, LINDEMAN GJ, HARRIS AW AND ADAMS JM. (1994). Cyclin D1 transgene impedes lymphocyte maturation and collaborates in lymphomagenesis with the myc gene. *EMBO J.*, **13**, 2124–32130.

BORG A, SIGURDSSON H, CLARK GM, FRENO M, FUQUA SAW, OLSSON H, KILLANDER D AND MCGURIE WL. (1991). Assocation of INT1/HST1 coamplification in primary breast cancer with hormone-dependent phenotype and poor prognosis. *Br. J. Cancer*, **63**, 136–142.

BUCKLEY MF, SWEENEY JE, HAMILTON JA, SINI RL, MANNING DL, NICHOLSON RI, DEFAZIO A, WATTS CKW, MUSHGROVE EA AND SUTHERLAND RL. (1993). Expression and amplification of cyclin genes in human breast cancer. *Oncogene*, **8**, 2127–2133.

CORNELIS RS, VAN VLIET M, VOS CBJ, CLETON-JANSEN A, VAN DE VIJVER MJ, PETERSE JL, MEERA KHAN P, BORRESEN A, CORNELISSE CJ AND DEVILEE P. (1994). Evidence for a gene on 17p13.3, distal to TP53, as a target for allele loss in breast tumours without p53 mutations. *Cancer Res.*, **54**, 4200–4206.

DAVIDOFF AM, KERNS BM, IGLEHART JD AND MARKS J. (1991). Maintenance of p53 alterations throughout breast cancer progression. *Cancer Res.*, **51**, 2605–2610.

EBINA E, STEINBERG SM, MULSHINE JL AND LINNOILA RI. (1994). Relationship of p53 overexpression and up-regulation of proliferating cell nuclear antigen with the clinical course of non-small cell lung cancer. *Cancer Res.*, **54**, 2496–2503.

ELSTON CW AND ELLIS OI. (1990). Pathology and breast cancer screening. *Histopathology*, **16**, 109–118.

FAILLE A, DE CREMOUX P, EXTRA JM, LINARES G, ESPIE M, BOURSTYN E, DE ROCQUANCOURT A, GIACCHETTI S, MARTY M AND CALVO F. (1994). p53 mutations and overexpression in locally advanced breast cancers. *Br. J. Cancer*, **69**, 1145–1150.

FANTL V, RICHARDS MA, LAMMIE GA, JOHNSTONE G, ALLEN D, GREGORY W, PETERS G AND BARNES DM. (1990). Gene amplification on chromosome band 11q13 and oestrogen receptor status in breast cancer. *Eur. J. Cancer*, **26**, 423–429.

FRYE RA, BENZ CC AND LIU E. (1989). Detection of amplified oncogenes by differential polymerase chain reaction. *Oncogene*, **4**, 1153–1157.

GILLETT C, FANTL V, SMITH R, FISHER C, BARTEK J, DICKSON C, BARNES D AND PETERS G. (1994). Amplification and overexpression of cylcin D1 in breast cancer detected by immunohistochemical staining. *Cancer Res.*, **54**, 1812–1817.

GREENWELL A, FOLEY JF AND MARONPOT RR. (1991). An enhancement method for immunohistochemical staining of proliferating cell nuclear antigen in archival rodent tissues. *Cancer Lett.*, **59**, 251–256.

HARRIS CC AND HOLLSTEIN M. (1993). Clinical implications of the p53 tumour suppressor gene. *N. Engl. J. Med.*, **239**, 1318–1328.

HENRY JA, HENNESSY C, LEVETT DL, LEMMARD TWJ, WESTLEY BR AND MAY FEB. (1993). Int-2 amplification in breast cancer: association with decreased survival and relationship to amplification of c-*erb*B2 and c-*myc*. *Int. J. Cancer*, **53**, 774–780.

HINDS PW, DOWDY SF, EASTON ENG, ARNOLD A AND WEINBERG R. (1994). A function of a human cyclin gene as an oncogene. *Proc. Natl Acad. Sci. USA*, **91**, 709–713.

KAPLAN EL AND MEIER P. (1958). Nonparametric estimation from incomplete observations. *J. Am. Stat. Assoc.*, **53**, 457–481.

KEYOMARSI K AND PARDEE AB. (1993). Redundant cyclin overexpression and gene amplification in breast cancer cells. *Proc. Natl Acad. Sci. USA*, **90**, 1112–1116.

LAMMIE GA AND PETERS G. (1991). Chromosome 11q13 abnormalities in human cancer. *Cancer Cells*, **3**, 413–420.

LEVINE AJ, PERRY ME, CHANG A, SILVER A, DITTMER DWU AND WELSH D. (1994). The role of the p53 tumour-suppressor gene in tumorigenesis. *Br. J. Cancer*, **69**, 409–416.

LONN U, LONN S, NILSSON B AND STENKVIST B. (1994). Breast cancer: significance of c-*erb*B2 and *int*-2 amplification compared with DNA ploidy, S-phase fraction, and conventional clinicopathological features. *Breast Cancer Res. Treat.*, **29**, 237–254.

LUKAS J, PAGANO M, STASKOVA Z, DRAETTA G AND BARTEK J. (1994). Cyclin D1 protein oscillates and is essential for cell cycle progression in human tumour cell lines. *Oncogene*, **9**, 707–718.

MANTEL N. (1966). Evaluation of survival data and two new rank order statistics arising in its consideration. *Cancer Chemother. Rep.*, **50**, 163–170.

MARCHETTI A, BUTTITTA F, PELLEGRINI S, CAMPANI D, DIELLA F, CECCHETTI D, CALLAHAN R AND BISTOCCHI M. (1993). p53 mutations and histological type of invasive breast carcinoma. *Cancer Res.*, **53**, 4665–4669.

MICHALIDES RJAM, VAN VEELEN N, HART A, LOFTUS B, WIENTJENS E AND BALM A. (1995). Overexpression of cyclin D1 correlates with recurrence in a group of 47 surgically resected squamous cell carcinomas of the head and neck. *Cancer Res.*, **55**, 975–978.

MOTOKURA T AND ARNOLD A. (1993). Cyclins and oncogenesis. *Biochim. Biophys. Acta*, **1155**, 63–78.

MUSHGROVE EA, HAMILTON JA, LEE CSL, SWEENEY KJE, WATTS CKV AND SUTHERLAND RL. (1993). Growth factor steroid and steroid antagonist regulation of cyclin gene expression associated with changes in T47D human breast cancer cell cycle progression. *Mol. Cell. Biol.*, **13**, 3577–3587.

QUELLE DE, ASHMUM RA, SHURTLEFF SA, KATO JY, BAR-SAGI D, ROUSSEL MF AND SHERR CJ. (1993). Overexpression of mouse D type cyclins accelerates $G_1$ phase in rodent fibroblasts. *Genes Dev.*, **7**, 1559–1571.

SCHUURING E, VERHOEVEN E, VAN TINTEREN H, PETERSE JL, NUNNINK B, THUNNISSEN FBJM, DEVILEE P, CORNELISSE CJ, VAN DE VIJVER MJ, MOOI WJ AND MICHALIDES RJAM. (1992*a*). Amplification of genes within the chromosome 11q13 region is indicative of poor prognosis in patients with operable breast cancer. *Cancer Res.*, **52**, 5229–5234.

SCHUURING E, VERHOEVEN E, MOOI WJ AND MICHALIDES RJAM. (1992*b*). Indentification and cloning of two overexpressed genes, U21B31/PRAD1 and EMS1, within the amplified chromosome 11q13 region in human carcinomas. *Oncogene*, **7**, 355–361.

SHERR CJ. (1993). Mammalian G1 cyclins. *Cell*, **73**, 1059–1065.

SILVESTRINI R, BENINI E, DAIDONE MG, VENEROI S, BORACCHI P, CAPPELLETTI V, DI FRONZO G AND VERONESI U. (1993). p53 as an independent prognostic marker in lymph node-negative breast cancer patients. *J. Natl Cancer Inst*, **85**, 965–970.

SUGARBAKER JP, GUNDERSON LL AND WITTES RE. (1985). Colorectal cancer. In *Cancer: Prinicples and Practices of Oncology*, De Vita VT, Hellmam S, Rosenberg SA. (eds) 2nd edn. pp. 800–803. Lippincott: Philadelphia.

THOR AD, MOORE DH, EDGERTON SM, KAWASAKI ES, REIHSAUS E, LYNCH HT, MARCUS JN, SCHWARTZ L, CHEN L, MAYALL BH AND SMITH HS. (1992). Accumulation of p53 tumour suppressor gene protein: an independent marker of prognosis in breast cancer. *J. Natl Cancer Inst.*, **84**, 845–855.

TSUDA H, HIROHASHI S, SHIMOSATO Y, HIROTA T, TSUGANE SD, YAMAMOTO H, MIYAJIMA N, TOYASHIMA K, YAMAMOTO T, YOKOTA T, YOSHIDA T, SAKAMOTO H, TERADA M AND SUGIMURA T. (1989). Correlation between long-term survival in breast cancer patients and amplification of two putative oncogene-coamplification units, *hst*-1/*int*-2 and c-*erb*B/*ear*-1. *Cancer Res.*, **49**, 3104–3108.

VOGELSTEIN B, FEARON ER, HAMILTON S, KERN SE, PREISINGER AC, LEPPERT M, NAKAMURA Y, WHITE R, SMITS AMM AND BOS JL. (1988). Genetic alterations during colorectal-tumour development. *N. Engl. J. Med.*, **319**, 525–532.

WANG TC, CARDIFF RD, ZUKERBERG L, LEES E, ARNOLD A AND SCHMIDT EV. (1994). Mammary hyperplasia and carcinoma in MMTV-cyclin D1 transgenic mice. *Nature*, **369**, 669–671.

WORLD HEALTH ORGANIZATION. (1981). *Histological Typing of Breast Tumours*, 2nd edn. World Health Organization: Geneva.